# Inequalities in Complementary Feeding Programs in Randomized Intervention and Nonintervention Areas after Program Implementation in Bangladesh, Ethiopia, and Vietnam

**DOI:** 10.1016/j.cdnut.2024.104426

**Published:** 2024-07-25

**Authors:** Tina G Sanghvi, Deepali Godha, Edward A Frongillo

**Affiliations:** 1 Alive & Thrive initiative, FHI 360, Family Health International, Washington, DC, United States; 2 Alive & Thrive initiative, FHI 360, Family Health International, Durham, NC, United States; 3 Consultant FHI 360, Indore, Madhya Pradesh, India; 4 Department of Health Promotion, Education, and Behavior, University of South Carolina, Columbia, SC, United States

**Keywords:** complementary feeding, socioeconomic inequality, counseling, community mobilization, agricultural extension, mass media, Bangladesh, Ethiopia, Vietnam, randomized controlled evaluations

## Abstract

**Background:**

Children in the 6–23-mo age group need to consume adequate energy and nutrients for healthy growth, brain development, cognition, and future productivity. Yet, large deficits remain. Complementary feeding practices can be improved on a large scale, but whether interventions reach and benefit disadvantaged mothers is not known.

**Objectives:**

To assess inequalities in complementary feeding practices and coverage following large-scale program implementation in 3 low- and middle-income countries.

**Methods:**

We re-analyzed evaluation data from randomized controlled studies conducted in Bangladesh, Ethiopia, and Vietnam and calculated socioeconomic inequality using Erreygers index for intervention and nonintervention areas. Intervention coverage indicators were developed in each country for interpersonal communication, community mobilization, agricultural extension, and media. We compared the direction and magnitude of inequalities in intervention and nonintervention areas.

**Results:**

At the endline, coverage, and practices related to complementary feeding were better in intervention areas, but coverage and practices favored the better-off and more educated mothers. In Bangladesh, only 5, and in Vietnam, only 1 out of 16 coverage variables measured favored disadvantaged mothers or were neutral; in Ethiopia, out of 18 coverage variables measured, 11 favored disadvantaged mothers or were neutral, and in all 3 countries, only 5–6 variables out of 16 that were measured favored disadvantaged mothers or were neutral.

**Conclusions:**

Inequalities exist both in how children in the 6–23-mo age group are fed and in programs aimed at improving complementary feeding practices. Programs favor the better-off and more educated mothers. We need to better understand context-specific program barriers and tailor targeted interventions to reach disadvantaged mothers.

## Introduction

Nutritional inadequacies in diets consumed during early childhood have significant impacts on the growth, health, cognition, and future productivity of individuals [1,2]. Breastfeeding alone is no longer sufficient after 6 mo of age, and WHO and UNICEF recommend age-appropriate feeding practices during 6–23 mo, including the age at which to introduce foods, types of foods, meal frequency, and amounts of foods [[3], [4], [5], [6]]. Despite widely available global and national guidelines, programs have not been successful in reducing the gaps between recommended and actual diets of young children [7]. Child malnutrition, including stunting, wasting, micronutrient deficiencies, and, more recently, overweight/obesity, tends to accelerate during this period of rapid growth [[8], [9], [10]].

Because of the substantial impact of young child nutrition on the future health and productivity of individuals, it is urgent that gaps in complementary feeding (CF) between the richer and poorer segments of the population are addressed to disrupt persisting poverty cycles in disadvantaged communities and low- and middle-income countries (LMICs) [1,8]. Surveys have documented socioeconomic disparities in infant and young child feeding practices and the quality of diets fed to infants and young children by wealth, education, and urban-rural residence [11]. The affordability of food sources containing a high concentration of energy and nutrient intake varies. UNICEF estimates that ∼139 million infants and young children globally consume less than the recommended 5 types of foods, and 31% consume a diet consisting of breast milk alone that does not meet requirements after 6 mo, accompanied only by starchy staples. A recent analysis found that the most affordable food sources of iron, zinc, calcium, vitamin A, folate, and vitamin B12 are liver, small fish, dark green leafy vegetables, milk, eggs, and, in some countries, ruminant meat, and groundnuts [12].

Several countries have identified factors associated with CF practices to define programming priorities for improving children’s diets [[13], [14], [15]]. A secondary analysis of socioeconomic inequalities in achieving a minimum acceptable diet in sub-Saharan Africa using 2010–2020 demographic and health survey data found that maternal education, media exposure, and household wealth were associated with better CF [16]. Systematic reviews that are based on controlled settings recommend using education interventions and combinations of food supplementation and education to reduce gaps between actual and recommended CF practices [[17], [18], [19]]. How these interventions would affect disadvantaged groups, however, is not known. The assessment of disparities in CF interventions implemented on a large scale through ongoing child health platforms has not been reported in the literature to our knowledge.

We previously published the impacts of 3 large-scale effectiveness trials that improved CF practices in Bangladesh, Ethiopia, and Vietnam [[20], [21], [22]]. Inequalities were not analyzed in these evaluations, and we do not know whether interventions reached and benefited the less wealthy and less educated mothers. The specific objective of this article is to assess inequalities in CF practices and program coverage by wealth and educational status of mothers in randomly assigned intervention and nonintervention areas in 3 large-scale programs.

## Methods

The programs varied from mostly community-based home visits for interpersonal communication (IPC) in Bangladesh [21] to the use of health extension workers (HEWs) and community volunteers for IPC in-home visits and health post visits combined with agricultural extension (AG) services for alleviating food access in Ethiopia [20], to a facility-based intervention model accompanied by promotion of facility visits in Vietnam [22]. In addition to health facility visits and home visits, the 3 countries promoted desirable CF norms using public education and mass media (MM) such as radio and television (TV) advertising and internet-based approaches in Vietnam and Bangladesh, mobile vans with loudspeakers and village theater in Ethiopia, and community meetings in Bangladesh and Ethiopia. The 3 programs are part of a large initiative (Alive & Thrive), and the study areas and evaluation clusters were randomly assigned as described in the impact articles on CF in Bangladesh [21], Ethiopia [20], and Vietnam [22]. The data were collected and analyzed by the International Food Policy Research Institute (IFPRI) to evaluate impacts; the methods and results have been published previously, as noted. The programs reached large-scale coverage through existing health services, AG services, community mobilization (CM) activities, and public education and MM channels. Systems strengthening was needed to prepare the delivery platforms such as through developing protocols, training, supervision, monitoring, and adaptive management with the use of data. Behavioral science was applied to understand and address barriers to the uptake of CF practices at the individual and family/community levels. For this article, we disaggregated the endline evaluation results by intervention and nonintervention areas and analyzed whether the inequality in program/intervention coverage and CF practices were different.

### Data sources

We extracted data from endline cross-sectional household surveys in Bangladesh and Vietnam conducted in 2014 and in Ethiopia in 2017. The study population was comprised of children in the age group 6–23.9 mo with final sample sizes of 1003 children in Bangladesh, 2720 in Ethiopia, and 1010 children in Vietnam. The sample size estimation was done separately in each country using demographic and health survey data to get baseline prevalence. Sampling was done to evaluate the impact on minimum dietary diversity (MDD) for children in the age group 6–23.9 mo. The literature on the effectiveness of behavior change interventions was used to obtain effect sizes. The original data collection was conducted for the impact assessment of the program interventions [23].

### Indicators

Indicators for CF practices are based on WHO recommendations [3,6,24]. Both CF practices and intervention coverage indicators are self-reported responses by mothers or fathers for some participant characteristics. In total, we assessed 8 variables for CF practices for inequality by wealth and the same variables for education status (total of 16) and 9 variables for CF program coverage for inequality by wealth and the same variables for education status (total of 18).

### CF practices

Food groups consumed included 5 food groups recommended by WHO: breast milk; animal source foods (ASFs) and dairy, including meat, fish, eggs, any seafood, and dairy products; plant source foods (PSFs), or fruits and vegetables; nuts, pulses, oilseeds include *daal*, beans, pea peanuts, groundnuts, and other nuts; and starchy staples including wheat, rice/porridge/gruel, noodles, and infant cereals. Timely introduction indicates if a child aged 6–8 mo is consuming solids, semi-solids, or soft foods. MDD indicates if the child had ≥5 of the following 8 food groups on the previous day or night: breast milk; dairy products (milk, yogurt, cheese); grains, roots, and tubers; vitamin A-rich fruits and vegetables; other fruits and vegetables; eggs; flesh foods (meat, fish, poultry, liver, or other organs); and legumes and nuts. Minimum meal frequency (MMF) is age-specific and indicates if a 6–8-mo-old child consumed ≥2 meals/snacks if still breastfeeding; a 9–23-mo-old child consumed ≥3 meals/snacks if still breastfeeding; or a 6–23-mo-old child consumed ≥4 meals/snacks if not breastfeeding.

### Intervention coverage

Four categories of interventions were analyzed individually: IPC or counseling, CM, MM or local media, and AG. The total number of interventions per mother was also analyzed.

The number of IPC visits was calculated from the number of times the mother visited at home or in the community or the number of times she visited a facility and was given advice on CF. In Bangladesh, IPC was delivered at home visits by 3 cadres of frontline workers; in Ethiopia, by HEWs in health posts and at-home visits by HEWs and volunteers; and in Vietnam, at primary health care centers by health providers. IPC content was measured using information on the number of messages/types of advice. Each country selected locally relevant messages and provided specific advice based on knowledge gaps and local contexts. In all countries, they included timing of introducing CF, diversity of foods, amounts, and number of meals by age, feeding a sick child, and continued breastfeeding to ≥2 y; in some countries, messages were also given on feeding style, e.g., responsive feeding or feeding patiently. Ethiopia also included allocating 1 chicken for eggs and a corner of the family farm for dark green leafy vegetables to be used for CF of the child.

The programs used different types of CM activities based on program needs and opportunities to engage existing community platforms. In Bangladesh, a score was given if CF was included in the following events and attended by the respondent, with a maximum score of 3: health forum, “popular theater” session, and video show presentation. In Ethiopia, this included participation in the following if CF was included, with a maximum score of 4: recall of printed illustrated messages (Child Nutrition Card), “Community Conversations” session, cooking demonstration, seeing or hearing a mobile van broadcasting “Sebat Mela” (a theater program that includes CF messages). In Vietnam, this included the number of times a respondent had participated in a village meeting where CF was discussed. The period of recall varied by country.

The types of public education or MM and recall periods for documenting exposure differed by context. In Bangladesh, this variable indicated having watched any of the nationally broadcast TV spots on CF or an animated film on CF. The binary indicator was conceptualized as 1 if the respondent had watched any of the TV advertisements or the film and 0 otherwise. In Ethiopia, this indicated the respondent hearing or seeing CF messages through any of multiple channels, some widely broadcast in the regional language and some targeted to intervention areas such as through local newspapers/magazines, radioTV, Sebat Mela program, poster/banner/board, local/community theater, loudspeaker, community event or gathering, and/or marketplace/shop. The binary indicator was conceptualized as 1 if the respondent had been exposed to any of the above and 0 otherwise. In Vietnam, this indicated seeing or hearing about CF in video clips on recommended CF practices or a clip urging mothers to seek counseling from a specially equipped franchised counseling center that was nationally and regionally broadcast such TV/radio or the internet combined with targeted approaches in intervention areas such as billboards, bus-wraps, and commune loudspeakers. This binary variable was a 1 if the respondent had seen or heard any of the above and 0 otherwise.

Only Ethiopia conducted AG activities, as household food insecurity was considered a major barrier to improving CF. The variable included having contact with an AG worker or development agent from the Department of Agriculture, where a CF-related topic was discussed.

The number of intervention platforms indicated exposure to multiple interventions and was categorized as any 1, 2, 3, or 4 platforms. It reflects the breadth and intensity of the programs and the ability to reach multiple recipients repeatedly through varied channels and formats.

### Participant characteristics

Child characteristics included the child’s age group (categorized as 6–8 mo; 9–11 mo; 12–23 mo), CF practices (such as timely introduction, MDD, and MMF), stunting, wasting, underweight, and a number of food groups consumed (categorized as 2 or less food groups, 3–4 food groups, and ≥5 food groups).

Maternal characteristics included mother’s age group (divided into 3 categories: <20 y, 20–30 y, and ≥30 y), mother’s occupation (categorized as agricultural work, housewife, or other), mother’s education (categorized as never attended school/class 1, completed classes 1–5, completed classes 6–9, completed classes 10–12 or higher), mother’s knowledge of CF practices (such as age of introduction of semisolid food; MMF or the correct knowledge of number of meals by age group; breastfeeding status, and knowledge about specific food groups).

Paternal and household characteristics included father’s occupation (categorized as farmer or other), household food insecurity (calculated as 4 categories: food secure, mild, moderate, severe as well as a score with a range of 0–27 where higher scores indicate higher food insecurity), and household dietary diversity (indicates the number of food groups consumed by anyone in the household other than the child).

### Statistical analysis

Inequality was measured across the beneficiary wealth and education spectrums in nonintervention and intervention areas. The study used Erreygers index (EI) for binary indicators and the concentration index for continuous measures to quantify the nature and extent of socioeconomic-related inequalities [25]. EI is independent of the prevalence of the indicator; it has been used previously and facilitated policy discussions on inequalities in private sector institutional delivery services in Nepal and Bangladesh [26]. These indices are derived from the Gini coefficient used for income inequality and are commonly used by economists. Inequality in nutrition has been analyzed previously using slope index of inequality and relative index of inequality, but these methods have limitations [27]. The EI and concentration index quantify the extent of inequality by assessing the distribution of the outcome of interest across the wealth spectrum (wealth inequality) or education spectrum (education inequality) [28]. EI and concentration index range between –1 to +1 where 0 indicates no inequality, −1 indicates that the outcome is concentrated among the worse off (poor/uneducated) or its distribution is pro-poor/pro-uneducated, and +1 indicates the concentration of the outcome among the better off (higher wealth categories/more highly educated) or the distribution is pro-better-off/pro-educated. The indices were estimated using the Stata “conindex” command, accounting for geographical clustering [29].

Although there are no standard cutoffs in the literature, small values of the indices (whether positive or negative) translate to negligible inequality, whereas high values in either direction indicate high inequality. Accordingly, index values for an indicator may be compared across countries or groups within a country to gain an understanding of which country or group has higher inequality. If 1 group shows a positive value and the other group shows a negative value, it provides further insight into a program’s reach and uptake. We have conducted statistical tests of the null of equality between nonintervention and intervention groups within each country using an F-test assuming equal variance across groups, and *P* values are reported.

For the wealth spectrum, the household wealth score was computed using principal component analysis of household assets and other aspects, whereas the education spectrum comprised the completed number of years of mothers’ education. Additionally, the prevalence of CF practices and CF program coverage by wealth quintiles was presented graphically to provide greater insights into inequalities in intervention and nonintervention areas. Analysis was conducted using Stata 15.1 [29]. Because of the evidence of a dose-response relationship with improved infant and young child feeding practices when mothers received multiple interventions [[30], [31], [32]], we examined the number of intervention exposures per mother.

Ethical approval was obtained by IFPRI from the institutional review boards of IFPRI and the designated countries for data collection, processing, storage, and use. No additional ethical reviews were conducted for secondary analysis of existing publicly available data.

## Results

The programs were implemented in varied contexts, as indicated by the characteristics of the study populations in Table 1. Almost two-thirds of the children in all countries belonged to the age group 12–23 mo, and the remaining were almost equally divided between 6–8 and 9–11 mo. Vietnam had substantially lower child undernutrition compared with other countries. The prevalence of stunting was highest in Ethiopia, whereas wasting was highest in Bangladesh. Education levels and occupations of mothers were markedly different, with over 90% of mothers in Vietnam having higher than sixth-class education, whereas in Ethiopia, two-thirds had never attended school or were in first grade. Three out of every 4 mothers were housewives in Bangladesh and Ethiopia, but <15% in Vietnam. Almost twice the proportion of fathers in Ethiopia were in agricultural occupations as compared with fathers in Bangladesh and Vietnam. More mothers in Ethiopia and Vietnam knew about the correct age for introducing semisolid or solid foods than in Bangladesh, whereas in Vietnam and Bangladesh, more mothers knew about ASF and PSF than in Ethiopia. Four of every 10 households were food insecure in Ethiopia and much less in Bangladesh and Vietnam. Overall, mothers in Ethiopia faced more food insecurity, less education, and more children under 2 y as compared to other countries.

**TABLE 1:** Characteristics of the study populations by country

Characteristics	Bangladesh		Ethiopia		Vietnam	
	Col %	*n*	Col %	*n*	Col %	*n*
Child’s characteristics						
Age 6–8 mo	18.5	186	19.5	531	17.9	181
Age 9–11 mo	16.8	169	20.4	555	16.9	171
Age 12–23 mo	64.6	648	60.1	1634	65.1	658
Undernutrition^1^						
Stunting	28.3	280	36.3	482	9.3	94
Wasting	18.9	188	16.0	212	3.2	32
Mothers’ characteristics						
Age group 18–25 y	10.9	109	36.0	978	35.2	356
Age group 26–35 y	72.7	729	51.2	1393	55.0	556
Age group 36–55 y	16.5	165	12.8	349	9.7	98
Occupation housewife	78.9	791	74.7	2031	14.4	145
Occupation agriculture	none	none	10.1	275	42.0	424
Occupation other	21.1	212	15.2	414	43.7	441
Education						
Never attended school/in class 1	14.1	141	63.4	1721	0.7	7
Completed class 1–5	30.6	307	14.6	397	6.4	65
Completed class 6–9	43.4	435	12.9	351	45.1	456
Completed class 10/higher	12.0	120	9.1	247	47.7	482
Mother with correct knowledge of CF						
Age to introduce SSF	55.9	561	84.2	2291	75.0	758
MMF or number of meals by age	46.7	468	98.4	2677	100	1010
Food groups that should be fed						
Starchy staples	64.2	644	72.8	1980	63.9	645
Animal source foods and dairy	96.3	966	76.5	2081	98.5	995
Plant source foods	87.8	881	55.8	1519	91.1	920
Household characteristics						
Fathers’ occupation, agriculture	28.0	281	67.9	1846	36.8	372
Fathers’ occupation, other	72.0	722	32.1	874	63.2	638
Number of children 6–23 mo						
0	0.0	0	-	-	0.0	0
1	100.0	1003	13.9	378	100.0	1010
≥2	-	-	86.1	2342	-	-
Wealth quintile						
Poorest	18.6	187	20.0	543	20.2	204
Poor	20.8	209	20.0	545	19.2	194
Middle	19.8	199	19.8	538	21.1	213
Rich	20.8	209	20.0	544	19.8	200
Richest	19.8	199	20.2	550	19.7	199
Food Insecurity						
Food secure	81.6	809	60.0	1623	74.8	744
Mild	4.9	49	9.4	254	7.9	79
Moderate	6.2	61	21.5	581	14.3	142
Severe	7.3	72	9.2	249	2.9	29

Abbreviations: CF, complementary feeding; MMF, minimum meal frequency; SSF, semisolid foods. Col% is the percent of the total sample measured for each characteristic, e.g., child's age group.

1 From Kim et al. [20].

Below, we summarize the overall differences at the endline between intervention and nonintervention areas in prevalence and inequalities of CF practices, followed by CF intervention coverage and inequalities in coverage. Finally, we illustrate inequalities among wealth quintiles using bar graphs.

### CF practices

In Bangladesh and Ethiopia, the prevalence of MDD among children in the 6–23-mo age group was higher in the intervention areas than in the nonintervention areas (Table 2). In Vietnam, MDD was high at 85% and 86% in both areas. In Bangladesh, the use of ASF was higher in intervention areas at the endline. PSF was higher in Bangladesh and Ethiopia in intervention areas at the endline. Nuts, pulses, and oilseeds were used more in intervention areas in Bangladesh and Vietnam as compared with nonintervention areas at the endline. Starchy staples were the most widely consumed food group in all study countries. The prevalence of breast milk consumption in the 6–23-mo age group was high at ≥95% in both nonintervention and intervention areas at the endline in Bangladesh and Ethiopia. Less than 70% of infants and young children consumed breast milk in Vietnam at the endline, and this did not differ across the treatment areas.

**TABLE 2:** Prevalence of complementary feeding practices by country and nonintervention compared with intervention areas

Indicators	Bangladesh %			Ethiopia %			Vietnam %		
	NI	I	*P* value	NI	I	*P* value	NI	I	*P* value
Timely introduction of foods	88.8	83.5	0.300	90.1	92.8	0.013	97.8	98.9	0.554
Children 6–23 mo with MDD	54.1	75.2	<0.001	15.4	22.1	<0.001	84.9	86.4	0.513
Food groups consumed									
Breast milk	94.6	97	0.061	97.9	98.5	0.313	63.1	68.0	0.102
Animal source foods, including dairy	78.9	88.6	<0.001	23.5	26.0	0.143	98.2	98.0	0.824
Plant source foods (fruits and vegetables)	77.1	88.4	<0.001	44.6	50.8	0.001	91.5	94.1	0.110
Nuts, pulses, and oilseeds	37.0	45.2	0.008	55.6	58.8	0.096	34.9	41.5	0.031
Starchy staples	94.6	97.2	0.040	87.1	91.5	<0.001	99.2	99.4	0.701

*P* values are given for differences in complementary feeding practices between nonintervention and intervention areas.

Abbreviations: I, intervention areas; MDD, minimum dietary diversity; NI, nonintervention areas

In Table 3, we examine whether inequalities were present in the recommended CF practices across wealth and education spectrums in intervention and nonintervention areas. Green-shaded cells indicate a pro-disadvantaged distribution of variables (minus sign >–0.003), red-shaded cells indicate a pro-advantaged distribution of variables (positive number >0.003), and no color in cells indicates a neutral distribution or almost equality was achieved (between –0.003 and 0.003). For categorizing meaningful numbers into pro-advantaged, pro-disadvantaged, or neutral, we chose.003 as the cutoff because it is close to 0.

**TABLE 3:**
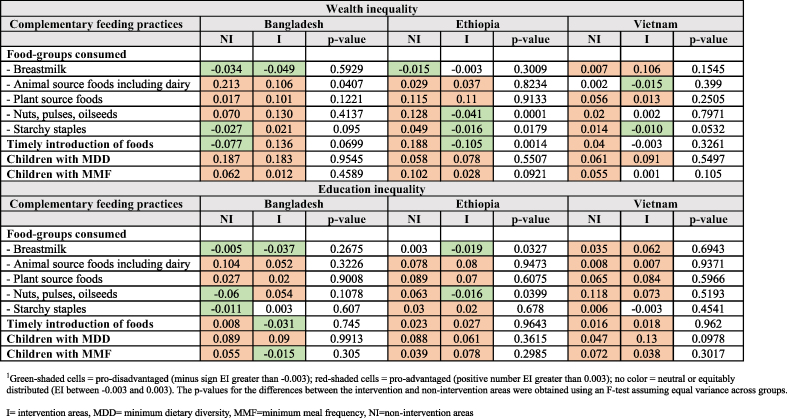
Inequalities in complementary feeding practices by country and nonintervention compared with intervention areas^1^

Wealth inequality in breast milk consumption among 6–23-mo-old infants and children was negligible except in intervention areas in Vietnam, where the practice was more common among children from better-off households. Similarly, high inequality was observed for consumption of ASF in the nonintervention areas and, to a smaller extent, in the intervention areas of Bangladesh, where it was common among the better-off households. There was a difference in the inequality for consumption of nuts, pulses, and oilseeds between nonintervention and intervention areas of Ethiopia. Timely introduction of foods was concentrated among the better-off households in the nonintervention areas of Ethiopia, whereas the opposite was true in the intervention areas. MDD was pro-rich in both areas across countries, except in intervention areas in Vietnam (neutral) of Bangladesh.

When inequality was calculated by the educational level of mothers, a difference was observed in the inequality for consumption of nuts, pulses, and oilseeds between nonintervention and intervention areas of Ethiopia.

### CF intervention coverage

Table 4 provides data on intervention coverage indicators in nonintervention and intervention areas across countries and indicates the different types of interventions used and the intensity or frequency of contacts with mothers by country and treatment groups. The countries varied in the type of intervention that reached the highest coverage, reflecting the strengths and limitations of program platforms and personnel responsible for delivering the programs. Overall, program coverage was high in all countries, and between 74% and 96% of mothers in the 3 countries received ≥1 type of program intervention in the intervention areas as compared with 55–75% in nonintervention areas. Coverage was higher in the number of IPC visits, number of IPC messages and coverage of community events in intervention as compared with nonintervention areas in the 3 countries. Public education/MM coverage was higher in intervention areas in Ethiopia and Vietnam but not Bangladesh; Bangladesh used national TV and radio that reached intervention and nonintervention areas, whereas the others used more localized channels or a mix of national and local channels. The percentage of mothers with exposure to any 2 interventions was greater in intervention as compared to nonintervention areas, with Bangladesh at 5% in nonintervention and 34% in intervention areas, Ethiopia at 26% nonintervention and 40% in intervention areas, and Vietnam at 6% nonintervention and 25% intervention areas.

**TABLE 4:** Complementary feeding intervention coverage by country and nonintervention compared with intervention areas

Interventions	Bangladesh		Ethiopia		Vietnam	
	NI	I	NI	I	NI	I
Number of IPC contacts in the last 6 mo (*P* ≤ 0.001 for all countries)						
≥1, %	7	71.8	17.6	39.9	9.4	35.3
None, %	93.0	28.2	82.4	60.1	90.6	64.7
1–3, %	4.2	11.8	12.3	26.5	8.2	26.8
4–24, %	2.8	60.0	5.3	13.4	1.2	8.5
Mean (SD)	0.24 (1.13)	5.69 (5.17)	0.51 (1.35)	1.27 (2.14)	0.17 (0.69)	0.97 (1.80)
Number of IPC messages/types of advice (*P* ≤ 0.001 for all countries)						
≥1, %	10.1	75.8	22.4	46.1	7.3	38.7
0, %	89.9	24.2	77.6	53.9	92.7	61.3
1–2, %	8.2	34.8	13.2	24.9	6.3	21.1
≥3, %	2.0	41.0	9.2	21.3	1.0	17.6
Mean (SD)	0.19 (0.66)	2.13 (1.70)	0.55 (1.30)	1.24 (1.75)	0.12 (0.48)	1.04 (1.60)
Community mobilization events (*P* ≤ 0.001 for all countries)						
≥1, %	1.0	18.4	58.1	92.3	4.4	9.7
0, %	99.0	81.6	41.9	7.7	95.6	90.3
1–2, %	1.0	18.4	58.1	92.3	4.4	9.7
Mean (SD)	0.01 (0.10)	0.20 (0.44)	0.67 (0.64)	1.59 (0.84)	0.04 (0.20)	0.17 (0.87)
Agricultural extension (*P* ≤ 0.001 for Ethiopia)						
Received a message about poultry (infant’s chicken) for CF, %	—	—	0	34.8	—	—
Received a message about farm DGLV (infant’s vegetable garden) for CF, %	—	—	0	39.1	—	—
Public education/mass media (*P* ≤ 0.001 for Ethiopia, *P* ≤ 0.05 for Vietnam)						
Recalled messages on CF, %	49.3	51.6	40.0	57.7	55.4	64.6
Total number of intervention platforms^1^ (*P* ≤ 0.001 for all countries)						
≥1, %	54.7	89.0	75.4	96.0	58.3	74.3
None, %	45.3	11.0	24.6	4.0	41.7	25.7
Any 1, %	49.3	43.6	39.0	24.6	50.8	42.7
Any 2, %	5.0	34.0	26.5	40.2	6.3	24.5
≥3, %	0.4	11.4	9.9	31.1	1.2	7.1
Mean (SD)	0.60 (0.60)	1.46 (0.84)	1.22 (0.95)	2.03 (0.92)	0.67 (0.65)	1.13 (0.88)

Abbreviations: CF, complementary feeding; DGLV, dark green leafy vegetables; I, intervention areas; IPC, interpersonal communication; NI, nonintervention areas.

1 Intervention platforms include interpersonal communication, community mobilization, agricultural extension, and public education/mass media.

Analysis of wealth inequality in program coverage (Table 5) shows the lower concentration of IPC contacts among the better-off households in intervention areas as compared to nonintervention areas of Ethiopia and Vietnam and close to neutral in Bangladesh (EI: 0.03). A similar pattern was seen in the number of IPC messages/advice recalled by mothers in Ethiopia. CM events in intervention areas favored more of the disadvantaged relative to nonintervention areas in Bangladesh and Ethiopia but not in Vietnam. MM favored the better-off households in all areas across countries, although the pro-better-off concentration was lower in intervention areas in Bangladesh compared with nonintervention areas, was almost the same for intervention and nonintervention areas in Ethiopia, and was more pro-better off in Vietnam in intervention areas. When all types of program interventions were considered, there was a higher concentration of exposure to any ≥1 interventions among the disadvantaged in intervention areas as compared to nonintervention areas in Bangladesh and Ethiopia, and almost no difference between nonintervention and intervention areas in Vietnam. Exposure to any 2 program interventions was concentrated among the better-off in all countries.

**TABLE 5:**
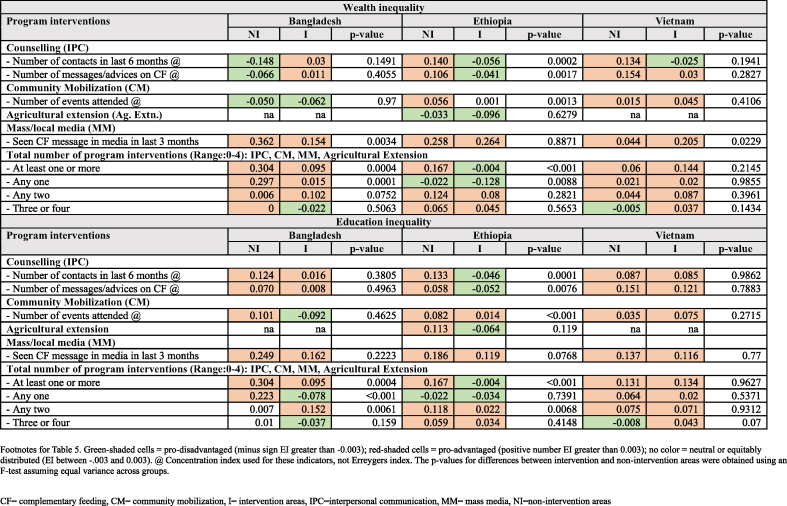
Inequalities in intervention coverage by country and nonintervention compared with intervention areas^1^

Inequality analysis by mothers’ educational level showed that inequality in coverage of most interventions was usually lower in the intervention compared with nonintervention areas but still favored the more educated mothers, except in Ethiopia. Inequality indices for exposure to any 1 intervention were lower in intervention areas as compared to nonintervention areas or were concentrated in the less educated in all countries. For any 2 program interventions, inequality was higher in intervention areas as compared to nonintervention areas of Bangladesh.

Among the number of coverage variables measured for inequality in intervention areas, 11 of 18 in Ethiopia favored disadvantaged mothers or were neutral, as compared with 5 of 16 in Bangladesh and 1 of 16 in Vietnam. Ethiopia and Vietnam showed larger differences in the number of variables related to pro-disadvantage inequalities in CF practices in intervention as compared with nonintervention areas at the endline as compared to Bangladesh. In Ethiopia, only 2 of 16, and in Vietnam, only 1 of 16 of variables that were measured for CF practices favored the disadvantaged mothers or were neutral in nonintervention areas, and in intervention areas of both countries, 6 of 16 favored disadvantaged mothers or were neutral. The intervention appeared to lessen inequalities even if they were not able to fully eliminate or reverse the pro-better-off bias. For example, the MM coverage EI indicator in Bangladesh was 0.362 in the nonintervention group but 0.154 in the intervention group, and in Vietnam, the concentration index for a number of CF messages in IPC was 0.154 in nonintervention and 0.03 in intervention areas, which suggest that there was a shift from more concentrated to less concentrated among the better-off mothers.

### CF practices across wealth quintiles

**FIGURE 1:**
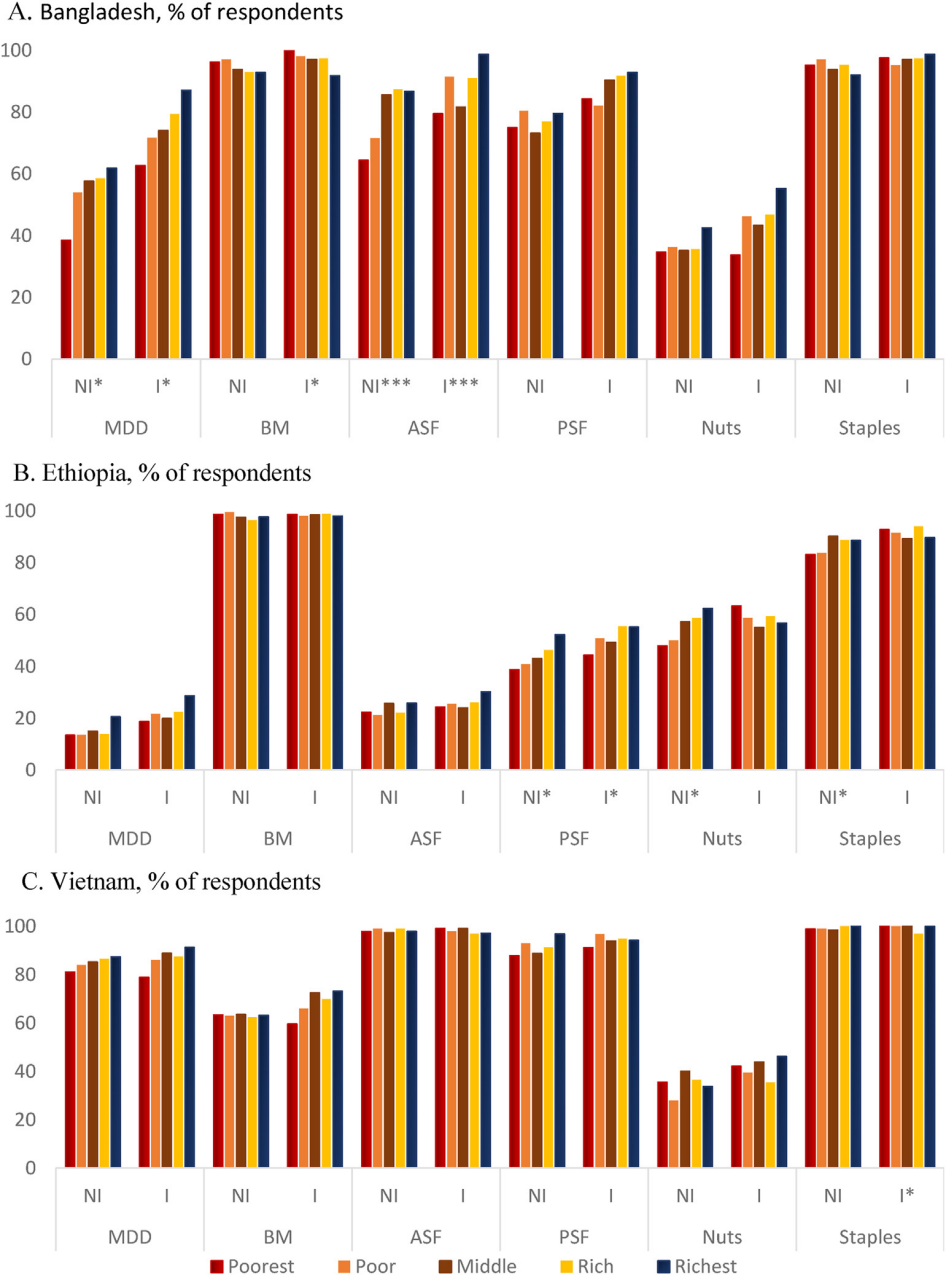
Complementary feeding practices across wealth quintiles by nonintervention areas (NI) and intervention areas (I). Differences across wealth quintiles are shown by ∗*P* < 0.05, ∗∗∗*P* < 0.001. ASF, animal source food; BM, breast milk; MDD, minimally adequate dietary diversity; PSF, plant source foods. Note: “Nuts” includes pulses and oilseeds. “Staples” refers to starchy staples.

Differences in CF practices by wealth quintiles in nonintervention and intervention areas are shown in Figure 1. In Bangladesh Figure1A, a stepwise rise in MDD can be seen in higher wealth quintiles and, to a certain extent, for ASF in nonintervention areas. Although the prevalence of MDD was higher in intervention areas, the wealth gradients remained for MDD. In Ethiopia Figure 1B, PSF and nuts, pulses, and oilseeds showed a gradient across wealth quintiles in nonintervention areas; it was less evident in intervention areas for PSF, whereas it showed a slight reversal for nuts, pulses, and oilseeds. In Vietnam Figure 1C, breast milk consumption after 6 mo was almost uniform across wealth quintiles, and consumption of nuts was high among the poorest and the middle class in nonintervention areas; in intervention areas, breast milk consumption improved among the better-off and nuts consumption was now highest among the richest and the middle class.

### Coverage of CF interventions across wealth quintiles

**FIGURE 2:**
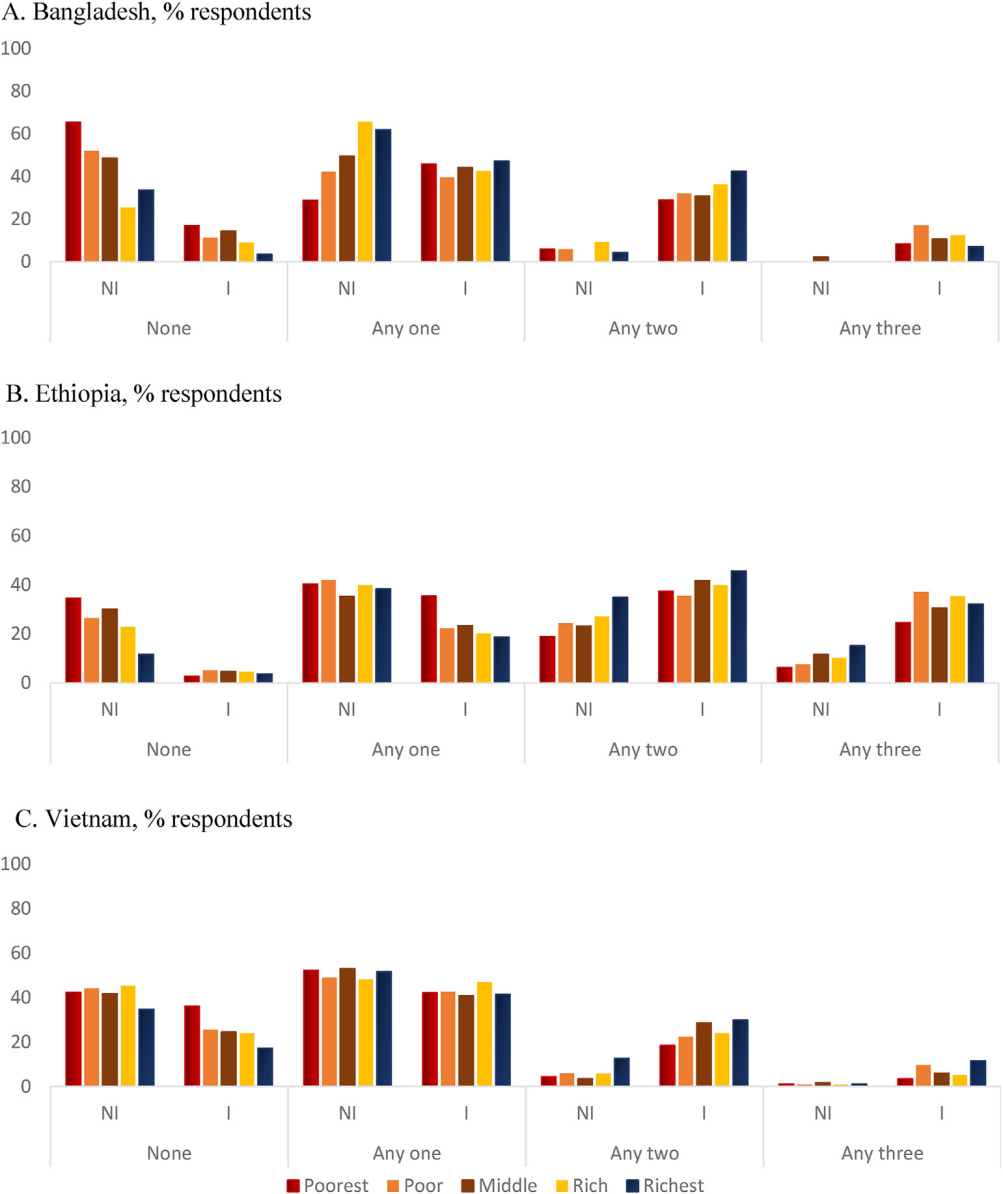
Program coverage as measured by the number of interventions that respondents were exposed to across wealth quintiles by nonintervention areas (NI) and intervention areas (I). The interventions included interpersonal communication, community engagement, and public education or mass media in all study countries, plus agricultural extension in Ethiopia.

Figure 2 shows CF intervention coverage by wealth quintiles as measured by the total number of different intervention exposures. In all 3 countries, there is a visible increase in intervention areas from a single intervention to ≥2 interventions. In Bangladesh Figure 2A, apart from the increase in percentages of mothers exposed to multiple interventions, the coverage shows a little more uniformity across wealth quintiles in intervention areas as compared to nonintervention areas. In Ethiopia Figure 2B, the exposure was higher for all categories in intervention areas, but more importantly, the poorest showed larger improvements. In Vietnam Figure 2C, as compared to nonintervention areas, exposure to a number of interventions was higher in intervention areas but was slightly higher among the richer households.

## Discussion

Inequalities in child undernutrition and deficits in the consumption of energy- and nutrient-rich foods in LMICs were already known [1,33,34]. This study provides evidence of persisting inequalities and wealth disparities in CF practices despite effective interventions that improved overall practices. This is the first study to our knowledge that provides information on the nature and magnitude of inequalities in large-scale CF programs in diverse LMIC country contexts. The results reveal that CF interventions were not consistently successful in reaching socioeconomically disadvantaged mothers nor in eliminating inequalities in their children’s CF practices. None of the countries used specific strategies to reduce inequalities, and this analysis suggests that in addition to designing programs for overall high coverage and impact on CF, countries like those in this study need to diagnose barriers and address them explicitly through targeted interventions to also reduce inequalities.

The types and levels of inequalities found in intervention areas at the endline differed in countries. For example, in Ethiopia, larger differences were documented in the number of CF practice and coverage variables that favored less advantaged mothers, whereas in Bangladesh and Vietnam, several indicators were less concentrated in the better-off women in intervention areas, indicating a shift toward equality. We cannot confirm what was specifically beneficial for equality in the programs due to a lack of data on associated factors. Other studies have found that both supply-related or program-delivery factors in health systems and demand factors in communities, families, and individuals may be involved [35,36]. Geographic accessibility, availability or convenience, financial limitations, and acceptability of services tended to reduce participation by the poor in health services [37], and key ingredients of success in reducing inequalities were found to be explicit and persistent efforts to reach the poor, pro-actively engaging the disadvantaged, locally adapted services, and ongoing monitoring of effects on the poor. A review of community health insurance programs identified the following demand factors: inadequate knowledge or awareness of the service/intervention, actual or perceived poor quality of the service offered, inconvenience due to time and effort needed, long distances to health facilities, negative provider attitude, and lack of belief in the benefit of the service [38]. In Ethiopia, services were provided through home visits and from community-based health posts by HEWs, health volunteers, and AG workers with greater proximity to participant families than in Vietnam, eg, that required primary health center visits; the program package in Ethiopia included explicit interventions to address access to food resources, unlike the other 2 countries.

Of the 16 variables measured for inequality in CF practices (8 for wealth and 8 for education), fewer favored disadvantaged mothers in Ethiopia’s nonintervention areas (2 of 16) as compared with intervention areas (6 of 16), and a similar pattern was seen in Vietnam, where 1 of 16 in nonintervention and 6 of 16 in intervention areas favored disadvantaged mothers. In Bangladesh, the difference between intervention and nonintervention areas was minimal (6 of 16 compared with 5 of 16). When wealth quintiles were disaggregated for CF practices, gradients in consumption of nutrient-rich foods remained clearly visible in Bangladesh and Vietnam across nonintervention and intervention areas, whereas lower gradients were detected in Ethiopia’s intervention areas as compared with nonintervention areas at the endline. The greater success of the Ethiopian CF program may be related to the inclusion of agriculture/food availability interventions in Ethiopia and greater potential for improvement in Ethiopia as indicated by the lowest proportion of children fed a diverse diet MDD and ASF and PSF foods in nonintervention areas as compared with Bangladesh and Vietnam. As reported in the impact evaluation publications, Ethiopia was the only country of the 3 included in this analysis that documented a reduction in stunting at endline [20].

Similarities were noted in CF feeding practices despite differences in sociocultural and economic characteristics of the study countries that are associated with what and how infants and young children are fed [39,40]. Our study noted some common elements across countries, such as the predominance of nutrient-poor starchy staples in young children’s diets, but there were variations in the use of beneficial food groups for CF. For example, ASF, PSF, and MDD were substantially higher in Vietnam as compared to Ethiopia. Our findings are consistent with a global review of region-specific differences in consumption of food groups by young children [7]; like our results, nuts, pulses, and oilseeds were consumed by a larger proportion of children in Africa (24%) as compared to Asia (18%).

UNICEF reported that >85 % of children 6–23 mo old in the 15 most impoverished countries, including Ethiopia, are lacking in nutrient-rich foods such as eggs, fish, poultry, meat, pulses, nuts, fruits, and vegetables and are mainly reliant on breast milk/dairy with starchy staples (grains, roots, and tubers) [34]. This study did not test for this, but based on the literature, it appears that the characteristics of the mothers, fathers, and households in our Ethiopian program areas may have placed them at greater likelihood of poor CF practices, whereas in Bangladesh, CF practices were more aligned with recommendations. For example, education among mothers in Bangladesh (14.1% with little or no schooling) as compared with Ethiopia (63.4% with little or no schooling) and better knowledge scores regarding the feeding of ASF and PSF to young children may have been beneficial for CF practices in Bangladesh. There was also greater food security and fewer children below 2 y in Bangladeshi households. Our results align with findings on wealth-related disparities in the consumption of nutrient-rich foods in India and global studies [7,41,42]. In India, Nguyen et al. [42] found that MDD and intake of iron-rich foods in the highest wealth quintiles was 2–4 times higher than in the lowest wealth quintiles. Globally, Choudhury et al. [7] showed that mothers’ education was a key determinant of child MDD, and mothers with ≥10 y of education were likely to feed their children 0.5 more food groups daily and were 13.3 pp more likely to achieve MDD. The low prevalence of breastfeeding in Vietnam among 6–23-mo-old children in our study is a reminder of CF vulnerabilities during rapid economic development.

Strategies for reducing inequities in public health programs have frequently recommended expanding and deploying community health workers (CHW), and recent reviews showed that CHWs successfully reach many marginalized groups, especially through home visits, but that health inequalities often persist due to limitations in the capacity of the disadvantaged to follow their advice [43,44]. The use of cash transfers and women’s support groups has also been recommended [45,46]. However, individual approaches such as cash transfers alone or the use of conditions may not be able to mitigate poverty and health inequalities in the presence of poor health services, and comprehensive strategies tailored to local needs are indicated [47]. The importance of ongoing advocacy, ideally accompanied by data to reveal the patterns of inequality and drive advocacy, has also been identified as a key component for bridging health equity gaps that require investments in health system strengthening, transportation infrastructure, and poverty alleviation, as well as better working conditions for CHWs and outreach workers [48]. Community-oriented programs, such as the Bangladesh and Ethiopia programs in this study, are noted as being targeted toward disadvantaged populations and regions and, therefore, although not achieving full equality, are likely to contribute to reducing inequalities [43]. But more needs to be done.

The limitations of our study include the use of self-reported recall information from mothers to estimate the prevalence of CF practices and program coverage. In all 3 countries, globally recommended indicators and methods were used, and the same data collection methods were used across countries [24]. Indicators in this article used for analyzing nutrition practices and coverage are proven to be associated with health and nutritional status outcomes [33,49]. The measurement and interpretation of inequality measures in nutrition practices and program coverage is a work in progress [50].

In the endline, although overall CF practices improved in our study countries, inequalities remained. Inequality indices calculated in our study suggest that program benefits for improving CF were unequal and favored the better-off and more educated groups. Bangladesh and Ethiopia, with somewhat better performances inequality of coverage, used multiple contact points and delivered services closer to the communities, e.g., IPC involved more cadres of trained workers, and they employed a broader array of CM strategies compared with Vietnam. Although Vietnam achieved high levels of MM exposure, facility visits needed for effective IPC did not reach the expected levels. The combined impact of interventions in all countries was important at an aggregate level but did not consistently reduce inequalities. Promising strategies to bridge the equality gaps indicated in this study include the use of existing large-scale government health posts and community-based services (Ethiopia), frontline worker networks of nongovernmental organizations (Bangladesh), and engaging >1 community-based sector such as AG (Ethiopia).

## Author contributions

The authors’ responsibilities were as follows – TGS, DG: designed research; DG, EAF, TGS: conducted research; DG: analyzed the data; TGS, DG, EAF: wrote the article; TGS: had primary responsibility for final content; and all authors: read and approved the final manuscript.

## Conflict of interest

The authors report no conflicts of interest.

## Data Availability

All datasets for the coverage data have been anonymized and are publicly available on the Harvard Dataverse repository at A&T's repository on Harvard Dataverse and https://dataverse.harvard.edu/dataverse/IFPRI.
